# Severe Dumping Symptoms Are Uncommon Following Transthoracic Esophagectomy But Significantly Decrease Health-Related Quality of Life in Long-Term, Disease**-**Free Survivors

**DOI:** 10.1007/s11605-020-04670-y

**Published:** 2020-11-04

**Authors:** F. Klevebro, P. R. Boshier, K. V. Savva, A. Waller, L. Hage, M. Ni, G. B. Hanna, Donald E. Low

**Affiliations:** 1 Department of Thoracic Surgery, Virginia Mason Medical Center, 1100 Ninth Ave, Seattle, WA 98101 USA; 2 Karolinska Institutet, Stockholm, Sweden; 3 Department of Surgery and Cancer, Imperial College London, London, UK

**Keywords:** Dumping symptoms, Esophagectomy, Health-related quality of life, Long-term follow-up

## Abstract

**Background:**

High-quality documentation of dumping symptoms after esophagectomy is currently limited. The aim of the study was to describe the incidence of symptoms associated with dumping syndrome and their relationship with health-related quality of life after esophagectomy.

**Methods:**

The study cohort was identified from prospective IRB-approved databases from two high-volume esophagectomy centers. Patients that were alive and without evidence of recurrence in April 2018 completed the validated Dumping Symptom Rating Scale and health-related quality of life questionnaires. Compound dumping symptom score was created by combining the individual scores for severity and frequency for each symptom.

**Results:**

In total, 171 patients who underwent esophagectomy 1995–2017 responded to the questionnaires, corresponding to a response rate of 77.0%. Median age was 66 years and median time from operation to survey was 5.5 years. Absent or mild problems in all nine dumping symptoms were reported by 94 (59.5%) patients; 19 (12.0%) patients reported moderate or severe problems in at least three symptoms, the most common being postprandial “need to lie down,” “diarrhea,” and “stomach cramps.” Increasing compound dumping symptom score was associated with significantly decreased function scores in all aspects of health-related quality of life except physical functioning (*P* < 0.005).

**Conclusions:**

Esophagectomy has the potential to change long-term eating patterns; however, the majority of patients in the study did not have severe postoperative dumping symptoms. On the other hand, moderate-to-severe dumping symptoms, which were reported by 12% of patients in this study, were strongly associated with decreased health-related quality of life.

## Introduction

Esophagectomy is often associated with significant postoperative gastrointestinal symptoms that adversely impact patients’ health-related quality of life (HRQOL).^1^,^2^ Recent improvement in survival of patients undergoing esophagectomy for cancer has increased the focus towards understanding and improving the long-term functional outcomes in this patient population.

The diagnosis of dumping syndrome can be defined as either “early” with symptoms occurring 10–30 min after a meal, or “late” with symptoms presenting 2–3 h after oral intake. Early dumping symptoms include abdominal pain, diarrhea, borborygmi, nausea, and bloating, as well as vasomotor symptoms including fatigue, a desire to lie down after meals, facial flushing, palpitations, perspiration tachycardia, hypotension, and syncope. Dumping syndrome diagnosis can be made with the use of symptom scoring systems or by an oral glucose challenge.^3^ The test is positive for dumping syndrome if the heartbeat increases by ≥ 10 beats per minute after intake of 50 g of glucose following a 10-h fast. Blood glucose and hematocrit are also measured, with a hematocrit increase of ≥ 3% in the first 30 min suggesting early dumping syndrome, and hypoglycemia 2–3 h after intake suggesting late dumping syndrome.^3^

Symptoms associated with dumping occur in 25–50% of patients who undergo gastric bypass with Roux-en-Y gastrojejunostomy reconstruction. In approximately 10% of patients, the condition is severe and persistent enough to warrant the diagnosis of dumping syndrome.^3^ The majority of patients who undergo esophagectomy will have postoperative gastrointestinal symptoms including early satiety and reflux. This is probably caused by exocrine pancreatic insufficiency and delayed gastric conduit emptying, as well as dumping.^1^,^4^,^5^ Studies assessing the incidence of dumping syndrome after esophagectomy are insufficient and use different classifications for the syndrome. The overall incidence of dumping symptoms after esophagectomy, according to a recent systematic review article, is approximately 20.2%, and even higher in patients with intraoperative pyloroplasty.^4^

This multi-center study aims to determine the incidence of early dumping symptoms and their effect on health-related quality of life in long-term disease-free survivors after esophagectomy.

## Methods

### Patients

Patients from two tertiary centers for surgical management of esophageal cancer were included in an observational and cross-sectional cohort study. The study cohort was identified from prospective institutionally approved databases, including treatment details of esophageal cancer patients. Eligible patients underwent esophagectomy with intrathoracic gastric tube reconstruction at Virginia Mason Medical Center, Seattle, USA (August 1995 to April 2017) or St. Mary’s Hospital, London, UK (February 2005 to May 2017). Inclusion criteria were age > 18 years, timeframe from surgery > 3 months, and disease-free status at the time of assessment. Patients were excluded if hospital records or institutional or national cancer registries showed disease recurrence or death. Patients with cognitive dysfunction, including dementia, were also excluded. Patients were recruited to participate in this study either at the time of routine outpatient clinical review or via telephone. This study was approved by the institutional review board, and patients were required to provide informed consent prior to participation in the manner approved by the local ethical boards reviewing this study.

Clinical data collected from patient records included age, gender, weight, comorbidities, details of neoadjuvant and adjuvant therapy, date of surgery, surgical approach, postoperative complications, clinical and pathological tumor stage, locations, and histology.

## Methodology for Questionnaires

Each patient that met the inclusion and exclusion criteria and was willing to participate completed a dumping score questionnaire and HRQOL questionnaires: (i) Dumping Symptom Rating Scale adopted from Laurenius et al.^6^, (ii) EORTC QLQ-30, (iii) EuroQol 5D, and (iv) SF-36. The Dumping Symptom Rating Scale assesses the severity and frequency of nine symptoms, occurring 10–30 min after eating, including fatigue, palpitations, sweating/flushing, cold sweats, a need to lie down, diarrhea, nausea/vomiting, stomach cramps, and fainting/“shaky feeling.” The scale also includes two questions concerning abdominal problems, faintness, or fatigue when drinking in relation to a meal or when consuming a heavily sweetened drink.

Patients were stratified according to the perceived severity of their symptoms: absent, mild, moderate, and severe. The frequency of symptoms was graded as follows: never, less than weekly, weekly, and daily. Adding the scores for severity and frequency (rated 0–3 for each) created a compound dumping symptom score. Questionnaires were distributed directly to subjects at the time of their routine follow-up clinic appointment or mailed to their home address (with provision for return mailing) or sent electronically via a purpose-designed online platform (REDCap).^7^

The EORTC QLQ C30 questionnaire was developed and validated by the European Organisation for Research and Treatment of Cancer (EORTC). The 30-item core questionnaire has nine multi-item scales measuring functions (global quality of life, physical, role, cognitive, emotional and social functioning) and symptoms (fatigue, pain, nausea and vomiting), and six single items measuring general cancer symptoms (dyspnea, appetite loss, insomnia, constipation, diarrhea, and financial impact).^8^ A higher score in the function scales indicates better function. In the symptom scales, a higher score indicates more symptoms. EuroQol 5D and SF-36 are validated and widely used HRQOL questionnaires.

## Statistical Analysis

Statistical analyses were performed using StataCorp 2015 (*Stata Statistical Software: Release 14*. College Station, TX: StataCorp LP). Chi-square and *T* tests were used for univariate comparisons. Linear regression analyses were performed to calculate mean score differences with 95% confidence interval (CI) for all HRQOL outcomes. Exploratory factor analysis was performed using SPSS (Ver. 24.0, IBM Corporation, Armonk, USA) to inform the grouping of component scores. Based on the analysis, the average scores for each of the factors identified were assessed. Linear regression was used to assess associations between these factors and the summative quality of life scores. Severity and frequency of each of the 9 symptoms were always in the same dimension.

## Results

In total, 222 eligible patients were contacted and agreed to participate in the study. Of these, 171 (77.0%) responded to the questionnaires and 159 (71.6%) completed all the included questionnaires. The study cohort underwent esophagectomy 1995–2017; median age was 66 years, and median time from operation to survey was 5.5 years (range 0.3–23.1). All esophagectomies were performed with an open technique; 85 (49.7%) patients had Ivor Lewis esophagectomy, 65 (38.0%) patients had left thoracoabdominal esophagectomy, of which 54 patients (83.1%) received anastomosis in the neck, 15 (8.8%) patients had three-stage esophagectomy (McKeown), 4 (2.3%) patients underwent transhiatal esophagectomy, and 2 (1.2%) patients were treated with total gastrectomy and lower esophagectomy. Intraoperative pyloroplasty was performed in 4 (2.3%) patients (Table 1).

**Table 1:** Patient characteristics

*N* (%)	*N* = 171
Age (range)	66.2 (30.0–90.0)
Time since surgery in years (range)	5.6 (0.3–23.1)
Gender	
Female	33 (19.3)
Male	138 (80.7)
ASA	
I	2 (1.2)
II	78 (48.5)
III	81 (50.3)
Unknown	10
Histological tumor type	
Adenocarcinoma	138 (80.7)
Squamous cell carcinoma	21 (12.3)
Other	7 (4.1)
Benign	5 (2.9)
Tumor location	
Proximal-middle esophagus	14 (8.3)
Distal esophagus	114 (67.9)
Gastroesophageal junction	35 (20.8)
Unknown	3
Benign	5 (3.0)
Neoadjuvant treatment	
None	62 (36.3)
Neoadjuvant chemotherapy	37 (21.6)
Neoadjuvant chemoradiotherapy	72 (42.1)
Surgical technique	
Two-stage esophagectomy (Ivor Lewis)	85 (49.7)
Left thoracoabdominal esophagectomy	65 (38.0)
Three-stage esophagectomy (McKeown)	15 (8.8)
Transhiatal esophagectomy	4 (2.3)
Total gastrectomy	2 (1.2)
Anastomosis location	
Thoracic	94 (55.3)
Cervical	76 (44.7)
Unknown	1
Pylorus	
Pyloroplasty	4 (2.3)

Continuous variables are presented as median and range. Values in parenthesis are percentages. *ASA*, American Society of Anesthesiology score

Absent or mild problems in all nine dumping symptoms were reported by 94 (60.0%) patients. In total, 19 (12.0%) patients reported moderate or severe problems in at least three symptoms. The most common severe problems were as follows: “a need to lie down,” “diarrhea,” and “stomach cramps” in 7.6%, 5.7%, and 5.1% of patients, respectively. Concerning symptomatic frequency, 60 (35.1%) patients reported at least one weekly or daily symptom rated moderate or severe, the most common being “fatigue” (*N* = 25, 15.8%), “a need to lie down” (*N* = 24, 15.2%), and “diarrhea” (*N* = 19, 12.0%). Due to symptoms of dumping, 97 (61.4%) patients reported that they avoided certain foods, the most common of which were fatty foods (*N* = 49, 31.0%), sugar-rich foods (*N* = 41, 25.9%), and dairy products (*N* = 38, 24.0%; Table 2).

**Table 2:** Dumping Symptom Rating scores in patients with long-term follow-up after esophagectomy

**Number of patients responding “yes” (%)** **Reflecting symptoms 10–30 min after eating**	**No problem**	**Minor/mild problems**	**Moderate problems**	**Severe/very severe problems**
Fatigue	66 (41.8)	66 (41.8)	18 (11.4)	8 (5.1)
Palpitations	123 (79.9)	23 (14.9)	5 (3.3)	3 (2.0)
Sweating/flushing	103 (66.0)	43 (27.6)	7 (4.5)	3 (1.9)
Cold sweats	124 (80.5)	24 (15.6)	4 (2.6)	2 (1.3)
A need to lie down	93 (59.2)	40 (25.5)	12 (7.6)	12 (7.6)
Diarrhea	84 (53.5)	53 (33.8)	11 (7.0)	9 (5.7)
Nausea/vomiting	101 (63.9)	43 (27.2)	7 (4.4)	7 (4.4)
Stomach cramps	96 (61.2)	41 (26.1)	12 (7.6)	8 (5.1)
Fainting/“shaky” feeling	100 (63.3)	42 (26.6)	13 (8.2)	3 (1.9)
Pain or vomiting when drinking during a meal	103 (67.3)	35 (22.9)	8 (5.2)	7 (4.6)
Heavily sweetened drink in the past week, *N* = 79				
Stomach discomfort, fatigue, or fainting when drinking a heavily sweetened drink	48/79 (60.8)	21/79 (26.6)	7/79 (8.9)	3/79 (3.8)
**Frequency**	**Never**	**Rarely**	**Weekly**	**Daily**
Fatigue	70 (45.6)	24 (15.7)	37 (24.2)	22 (14.4)
Palpitations	119 (80.4)	10 (6.8)	16 (10.8)	3 (2.0)
Sweating/flushing	105 (70.5)	18 (12.1)	21 (14.1)	5 (3.4)
Cold sweats	125 (82.8)	11 (7.3)	12 (8.0)	3 (2.0)
A need to lie down	82 (54.0)	19 (12.5)	34 (22.4)	17 (11.2)
Diarrhea	77 (51.0)	21 (13.9)	39 (25.8)	14 (9.3)
Nausea/vomiting	96 (64.0)	21 (14.0)	24 (16.0)	9 (6.0)
Stomach cramps	87 (58.0)	20 (13.3)	32 (21.3)	11 (7.3)
Fainting/“shaky” feeling	93 (61.2)	21 (13.8)	31 (20.4)	7 (4.6)
**Compound dumping symptom score, median (IQR) 8.0 (2.0–16.0)**				
	**Yes**	**No**		
Avoid certain food	97 (61.4)	61 (38.6)		
Fatty food	49 (31.0)	109 (69.0)		
Meat products	27 (17.1)	131 (82.9)		
High-fiber foods	18 (14.4)	140 (88.6)		
Fruits	8 (5.1)	150 (94.9)		
Sugar-rich products	41 (25.9)	117 (74.1)		
Raw vegetables	18 (14.4)	140 (88.6)		
Milk or dairy products	38 (24.0)	120 (76.0)		
Other food	50 (31.6)	108 (68.4)		

Increased compound dumping symptom score was associated with significantly decreased function scores in all aspects of HRQOL, except physical functioning, when measured with EORTC QLQ C30 as well as SF36 (*P* < 0.05). There was also a statistically significant association between all measured symptoms on EORTC QLQ C30 and increased compound dumping symptom score. Nausea and vomiting, fatigue, and diarrhea had the strongest correlation to increased dumping symptom score (Table 3).

**Table 3:** Linear regression of HRQOL scores and association with compound dumping symptom score in patients after esophagectomy

EORTC QLQ-Core30		*P* value
Function scores (higher score indicates better function)		
Global health status	− 0.81 (− 1.12 to − 0.52)	< 0.001
Physical functioning	− 0.26 (− 0.58 to 0.06)	0.118
Role functioning	− 0.69 (− 1.08 to − 0.31)	< 0.001
Emotional functioning	− 0.89 (− 1.21 to − 0.56)	< 0.001
Cognitive functioning	− 0.92 (− 1.24 to − 0.59)	< 0.001
Social functioning	− 1.22 (− 1.61 to − 0.83)	< 0.001
Symptom scores (higher score indicates more symptoms)		
Fatigue	1.03 (0.68 to 1.37)	< 0.001
Nausea and vomiting	1.24 (0.97 to 1.52)	< 0.001
Pain	0.90 (0.46 to 1.34)	< 0.001
Dyspnea	0.96 (0.57 to 1.36)	< 0.001
Insomnia	0.69 (0.22 to 1.16)	0.004
Appetite loss	0.96 (0.48 to 1.45)	< 0.001
Constipation	0.61 (0.19 to 1.03)	0.005
Diarrhea	1.18 (0.77 to 1.58)	< 0.001
Financial difficulties	0.36 (− 0.06 to 0.78)	0.093
SF-36 (higher score indicates better function)		
Physical functioning	− 0.15 (− 0.58 to 0.27)	0.481
Role limitations due to physical health	− 0.83 (− 1.49 to − 0.16)	0.016
Role limitations due to emotional problems	− 0.96 (− 1.54 to − 0.39)	0.001
Energy/fatigue	− 0.84 (− 1.19 to − 0.49)	< 0.001
Emotional well-being	− 0.85 (− 1.15 to − 0.55)	< 0.001
Social functioning	− 0.96 (− 1.31 to − 0.61)	< 0.001
Pain	− 0.85 (− 1.30 to − 0.40)	< 0.001
General health	− 1.04 (− 1.35 to − 0.73)	< 0.001
EuroQol 5D (higher score indicates more problems)		
Mobility	0.01 (− 0.01 to 0.02)	0.305
Self-care	0.01 (0.0 to 0.02)	0.028
Usual activities	0.03 (0.02 to 0.04)	< 0.001
Pain/discomfort	0.03 (0.02 to 0.05)	< 0.001
Anxiety/depression	0.04 (0.02 to 0.05)	< 0.001
Today’s health on a 0–100 scale	− 0.74 (− 0.98 to − 0.49)	< 0.001

Higher age was associated with a statistically significant decrease in median compound dumping symptom score of − 0.24 (95% confidence interval − 0.5 to − 0.0, *P* = 0.030) per year. Malignant indication for esophagectomy was associated with decreased median compound dumping syndrome score compared with benign esophagectomy of − 16.0 (95% confidence interval − 25.7 to − 6.3, *P* = 0.001). Intraoperative pyloroplasty was only used in four patients in the study; there was no significant difference in compound dumping symptom score after pyloroplasty compared with other patients. There was no association with gender, ASA score, neoadjuvant treatment, time from surgery, surgical technique, tumor location, or anastomosis location (Table 4).

**Table 4:** Quantile regression of clinical and treatment factors and association with compound dumping symptom score in patients after esophagectomy

	Median change	*P* value
Age	− 0.2 (− 0.5 to− 0.0)	0.030
Time from surgery	0.1 (− 0.4 to 0.6)	0.717
Female vs. male (ref.)	4.0 (− 0.7 to 8.7)	0.095
American Society for Anesthesiology score*	− 3.0 (− 6.4 to 0.4)	0.081
Malignant vs. benign (ref.) indication	− 16.0 (− 25.7 to − 6.3)	0.001
Squamous histology vs. adenocarcinoma (ref.)	4.0 (− 1.9 to 9.9)	0.185
Neoadjuvant treatment vs. surgery alone (ref.)	− 2.0 (− 4.1 to 0.1)	0.064
Surgical technique**	0.9 (− 0.8 to 2.8)	0.261
Pyloroplasty vs. no intervention (ref.)	− 2.0 (− 12.1 to 8.2)	0.699
Tumor location (proximal vs. lower or GEJ)	1.8 (− 2.5 to 4.5)	0.568
Neck vs. chest anastomosis (ref.)	2.1 (− 2.18 to 6.18)	0.346

*American Society for Anesthesiology Score I, II or III.

**Two-stage esophagectomy (Ivor Lewis), left thoracoabdominal esophagectomy, three-stage esophagectomy (McKeown), transhiatal esophagectomy, or other techniques

Factor analysis determined that the dumping syndrome scale could be classified into five distinct dimensions, which were general symptoms, hemodynamic symptoms, gastrointestinal symptoms, palpitations, and diarrhea. Of these five factors, gastrointestinal symptoms (nausea/vomiting, stomach cramps, and pain or vomiting during meal) had the largest and most reproducible impact on the three summative HRQOL score that were assessed (*P* < 0.01).

## Discussion

This study included a cohort of short- and long-term survivors after esophagectomy from two high-volume centers for esophageal surgery. The results show that the majority of patients do not report problems with dumping symptoms after esophagectomy. The study does demonstrate that, in a study population in which pyloric intervention was uncommon, 12% of the patients report moderate or severe problems with at least three dumping symptoms. This number is lower than previously reported; however, the prevalence of dumping symptoms after esophagectomy has not been studied with clear definitions, which makes comparisons with other studies difficult.^4^ Furthermore, both compound dumping symptom score and the dimension of gastrointestinal symptoms that was identified by factor analysis were strongly associated with decreased HRQOL. The technical evolution of esophagectomy needs to identify factors to limit the incidence of postoperative dumping symptoms to improve long-term gastrointestinal function and HRQOL for patients after esophagectomy.

Strengths of the study include the relatively large study cohort and long-term follow-up, with patients undergoing esophagectomy up to 23 years prior to enrollment, and median time from surgery of 5.6 years. The response rate was high, reducing the risk for selection bias. Patients responded to the validated Dumping Symptom Rating Scale^6^ and widely used and validated HRQOL questionnaires. Study limitations include the lack of one clear definition for dumping syndrome after esophagectomy, and that reported symptoms were not validated with a glucose stress test.^3^ All operations in the study were performed with an open technique, which makes the conclusion primarily applicable to open esophagectomy patients. However, there are no studies indicating that minimally invasive technique is associated with a significant change in conduit function, but this issue needs further attention in future studies.^9^

The prevalence of dumping syndrome after esophagectomy has been described from 0 to 78% in the literature, with an average of 20.2% of patients reporting dumping symptoms in a systematic review article.^4^ The heterogeneous definition of dumping syndrome makes epidemiological comparisons difficult. Dumping symptoms were shown to reduce with age in the current study, which has been previously demonstrated.^10^,^11^ Time from surgery was, however, not associated with decreased symptoms.

The role of pyloroplasty remains controversial in surgery for esophageal cancer. Pyloroplasty has been reported to be associated with an increased risk for dumping syndrome.^12^ In a retrospective study in which 83% of patients had intraoperative pyloroplasty or pyloromyotomy, 50% reported postoperative dumping symptoms.^11^ A retrospective study demonstrated a statistically non-significant trend for increased dumping syndrome after pyloromyotomy or pyloroplasty.^13^ The result of a review article from 2001 concluded that, based on scientific evidence, pyloroplasty or pyloromyotomy should not be applied as a standard in esophagectomies with gastric tube reconstruction.^14^ Postesophagectomy care should consider including increased monitoring, and objective testing for postoperative dumping syndrome, and initiating of dietary or medical intervention when testing is positive. Standardized construction of a narrow vertical gastric conduit with an anastomosis either in the neck or high in the chest, rather than pyloric intervention, may decrease the incidence of long-term dumping symptoms and improve HRQOL.

In conclusion, this study shows that early dumping symptoms are associated with significantly decreased HRQOL and, although severe problems are relatively uncommon, the majority of patients in the study reported gastrointestinal symptoms and decreased HRQOL compared with the general population.^15^ Increased focus on patient-reported outcomes of gastrointestinal symptoms and HRQOL is warranted in future prospective studies. Functional outcomes after esophagectomy, especially gastrointestinal symptoms, associated with dumping syndrome can be improved to increase HRQOL and survivorship for patients after esophagectomy.
